# Zoledronic acid prevents pagetic-like lesions and accelerated bone loss in the p62^P394L^ mouse model of Paget's disease

**DOI:** 10.1242/dmm.035576

**Published:** 2018-08-23

**Authors:** Anna Daroszewska, Lorraine Rose, Nadine Sarsam, Gemma Charlesworth, Amanda Prior, Kenneth Rose, Stuart H. Ralston, Robert J. van ‘t Hof

**Affiliations:** 1 Department of Musculoskeletal Biology, Institute of Ageing and Chronic Disease, University of Liverpool, Liverpool L7 8TX, UK; 2 Rheumatic Diseases Unit, University of Edinburgh, Edinburgh EH4 2XU, UK

**Keywords:** Paget's disease of bone, Genetic animal models, Ageing, Bone morphometry, Antiresorptives, Zoledronic acid

## Abstract

Paget's disease of bone (PDB) is an age-related metabolic bone disorder, characterised by focally increased and disorganised bone remodelling initiated by abnormal and hyperactive osteoclasts. The germline P392L mutation of *SQSTM1* (encoding p62) is a strong genetic risk factor for PDB in humans, and the equivalent mutation in mice (P394L) causes a PDB-like disorder. However, it is unclear why pagetic lesions become more common with age. Here, we assessed the effect of the p62 P394L mutation on osteoclastogenesis and bone morphometry in relation to ageing, the natural history of lesion progression in p62^P394L^ mice and the effect of zoledronic acid (ZA) on lesion development. p62^P394L+/+^ osteoclast precursors had increased sensitivity to RANKL (also known as TNFSF11) compared with wild-type (WT) cells, and the sensitivity further increased in both genotypes with ageing. Osteoclastogenesis from 12-month-old p62^P394L+/+^ mice was twofold greater than that from 3-month-old p62^P394L+/+^ mice (*P*<0.001) and three-fold greater than that from age-matched WT littermates. The p62^P394L+/+^ mice lost 33% more trabecular bone volume in the long bones by 12 months compared with WT mice (*P*<0.01), and developed pagetic-like lesions in the long bones which progressed with ageing. ZA prevented the development of pagetic-like lesions, and increased trabecular bone volume tenfold compared with vehicle by 12 months of age (*P*<0.01). This demonstrates that ageing has a pro-osteoclastogenic effect, which is further enhanced by the p62 P394L mutation, providing an explanation for the increased penetrance of bone lesions with age in this model. Lesions are prevented by ZA, providing a rationale for early intervention in humans.

## Abstract (precis)

**Summary:** We show that age-related increased osteoclastogenesis is potentiated by the p62 P394L mutation, which allows for development of pagetic-like lesions that progress with ageing, but are prevented by zoledronic acid.

## INTRODUCTION

Paget's disease of bone (PDB) is the second most common metabolic bone disorder after osteoporosis and its prevalence increases with ageing. PDB is characterised by focally increased and disorganised bone remodelling, which leads to the formation of poor-quality woven bone. Consequently, pain, bone expansion, deformities, secondary osteoarthritis, pathological fractures and, very rarely, sarcoma can develop in the skeletal sites affected by PDB (Ralston et al., 2008).

Pagetic lesions are believed to be initiated by abnormal, enlarged, hypernucleated and hyperactive osteoclasts, which drive the progression of osteolysis, with increased and disorganised osteoblast-mediated new bone formation leading to the production of woven bone. Bisphosphonates (BPs) have been used for decades as treatment for PDB owing to their ability to suppress osteoclast activity, and heal lytic lesions with subsequent restoration of histologically normal new bone deposition and symptomatic improvement (Meunier and Vignot, 1995; Reid and Hosking, 2011; Reid et al., 1996). Currently, the most potent of the BPs, zoledronic acid (ZA), is the treatment of choice for PDB as it effectively suppresses bone remodelling and typically normalises bone turnover markers, in some patients for up to 10 years (Cundy et al., 2017). There is evidence that BPs improve pain (Corral-Gudino et al., 2017); however, intensive treatment of established PDB does not improve disease outcome in terms of quality of life measures or pain (Langston et al., 2010; Tan et al., 2017), which implies that preventive intervention, prior to lesion development, could be a better strategy for susceptible individuals.

It has been shown that genetic predisposition to PDB is mediated through the effects of several common predisposing variants of moderate effect size coupled with influence of rare variants, which have large effect size (Albagha et al., 2010, 2013). The most important predisposing gene for PDB is sequestosome 1 (*SQSTM1*), which encodes p62, a scaffolding protein involved in a variety of cellular processes including signalling and protein degradation (Rea et al., 2013). PDB-associated mutations of p62 tend to cluster in (but are not limited to) the ubiquitin-associated domain and occur in up to 40% of patients with a family history of PDB and 5-10% of patients with ‘sporadic’ disease (Hocking et al., 2002; Laurin et al., 2002). The most common and most studied PDB-associated p62 mutation is P392L (Hocking et al., 2002; Laurin et al., 2002). Although it is not entirely clear how p62 mutations cause or predispose to PDB, current evidence points to increased receptor activator of nuclear factor kappa-B ligand (RANKL; also known as TNFSF11)-mediated signalling enhancing osteoclastogenesis and dysregulated protein degradation (Rea et al., 2013).

The penetrance of PDB in p62 mutation carriers rises with age to reach between 80% and 90% by the seventh decade of life (Morissette et al., 2006). However, recent observations of a delayed disease onset in the p62 mutation carriers’ offspring, a less severe disease phenotype (Bolland et al., 2007; Cundy et al., 2015) and decreasing incidence of PDB in many countries over the past 25 years (Corral-Gudino et al., 2013; Cundy et al., 1997) suggest that nongenetic or environmental factors might play a role in triggering the disease and/or affecting its severity. The historical viral hypothesis is controversial owing to conflicting evidence (Friedrichs et al., 2002; Rima et al., 2002; Visconti et al., 2017); nevertheless, transgenic mouse models of PDB-like disorders induced by viral sequences have been reported, suggesting that overexpression of measles virus nucleocapsid protein in osteoclast precursors increased osteoclast activity and bone resorption *in vitro* and induced areas of high bone turnover in the vertebrae with a 30% penetrance at 12 months of age (Kurihara et al., 2006a,b). One group did not detect evidence of high bone turnover with the characteristics of PDB in the vertebrae of mice bearing a knock-in p62 P394L mutation (equivalent to the human P392L mutation) (Hiruma et al., 2008).

We reported that although the p62 P394L mutation seldom causes vertebral lesions in mice, it frequently causes PDB-like lesions in the long bones, which become increasingly penetrant with ageing (Daroszewska et al., 2011). However, the mechanisms responsible for the age-related increase in penetrance remain unclear and there have been no studies on whether or not BPs could modify this phenotype.

Here, we revisit the p62^P394L^ model of PDB and seek to validate it in the context of age-related osteoclastogenesis. We explore the ‘natural history’ of murine pagetic-like lesion evolution and relate it to human pagetic lesion progression. Finally, we investigate the role of ZA in prevention of the PDB-like phenotype.

## RESULTS

### Osteoclast formation increases in p62^P394L^ mice with ageing

**Fig. 1.**
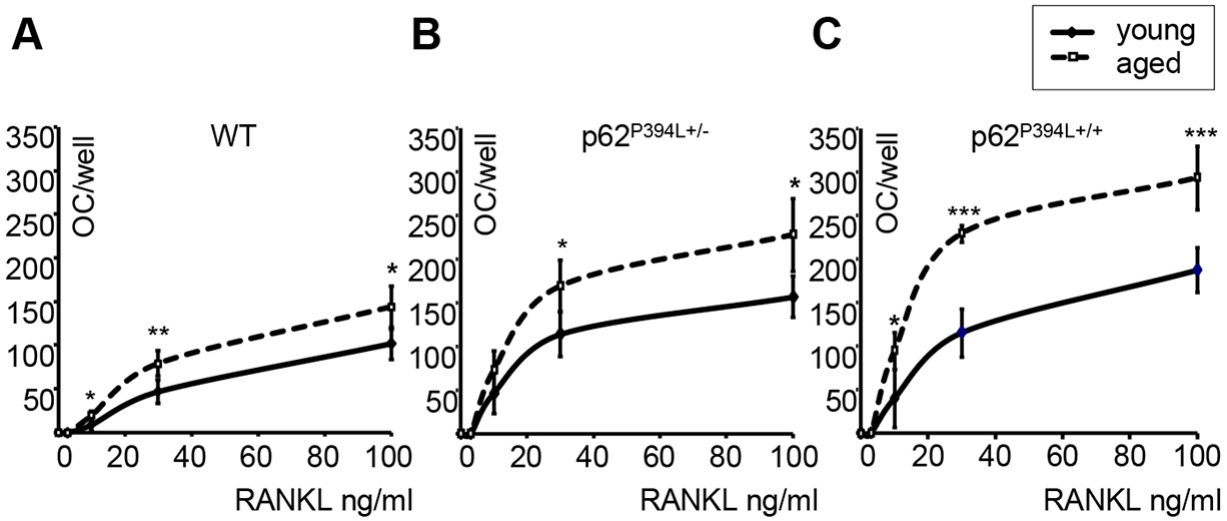
**Osteoclast formation is increased in p62^P394L^ mice with ageing.** (A-C) Quantitation of osteoclast (OC) numbers in M-CSF- and RANKL-stimulated macrophage cultures from young adult (3-month-old) and aged (12-month-old) wild-type (WT; A), p62^P394L+/−^ (B) and p62^P394L+/+^ (C) mice. RANKL stimulation at 0, 3, 10, 30 and 100 ng/ml. Data are mean±s.d. from three independent experiments. *P<0.05, **P<0.01, ***P<0.001 (one-way ANOVA).

Studies *in vitro* showed that macrophage colony-stimulating factor (M-CSF)- and RANKL-induced osteoclast formation from bone marrow-derived macrophages was significantly greater in aged (12-month-old) WT mice when compared with young adult (3-month-old) WT mice (Fig. 1A). The number of osteoclasts generated from young adult p62^P394L+/−^ mice was significantly greater when compared with young adult WT littermates, whereas the number of osteoclasts generated from aged p62^P394L+/−^ mice was greater when compared with young adult and aged WT mice (Fig. 1A,B). This effect was even more striking in the p62^P394L+/+^ mice. The number of osteoclasts generated from aged p62^P394L+/+^ mice increased approximately twofold when compared with young adult p62^P394L+/+^ mice (Fig. 1C) and threefold when compared with aged WT littermates (Fig. 1A,C). Moreover, osteoclast precursors from p62^P394L+/+^ mice showed evidence of increased sensitivity to RANKL as compared with WT cells, at 10 ng/ml, 30 ng/ml and 100 ng/ml RANKL stimulation, which was intensified by ageing (Fig. 1C). A similar effect was seen in osteoclast precursors generated from the p62^P394L+/−^, although not as pronounced as in the homozygotes (Fig. 1B). Thus, ageing increases RANKL-induced osteoclastogenesis, and the p62 P394L mutation further enhances the age-related increase in osteoclastogenesis with a gene dosage effect.

### The p62^P394L^ mutation is associated with accelerated age-related long bone loss

**Fig. 2.**
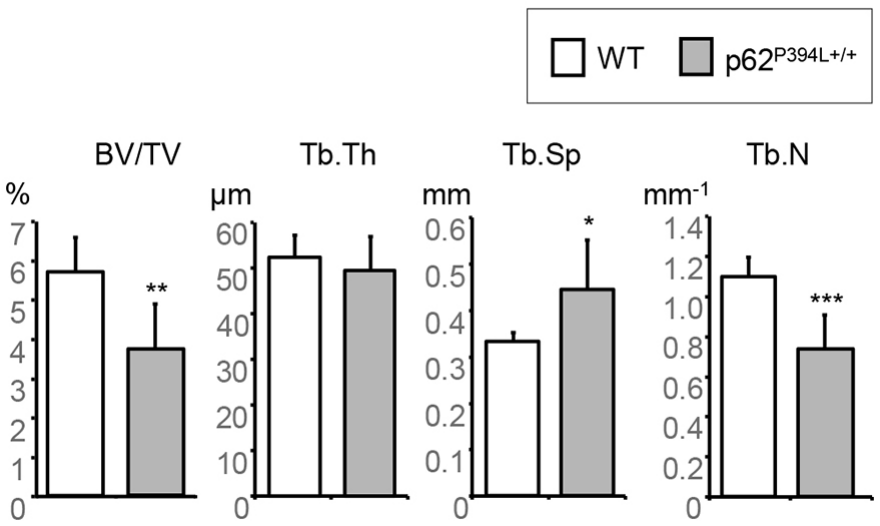
**p62^P394L+/+^ mice show accelerated bone loss in the long bones with ageing.** Distal femurs of 12-month-old female p62^P394L+/+^ (*n*=7) and WT (*n*=6) mice were scanned *ex vivo* with μCT at 4.5 μm resolution. BV/TV, bone volume per tissue volume; Tb.Th, trabecular thickness; Tb.Sp, trabecular separation; Tb.N, trabecular number. Data are mean±s.d. *P<0.05, **P<0.01, ***P<0.001 (Student's *t*-test).

We have previously shown that there was no significant difference in trabecular bone density and structure at the proximal tibial metaphyses of young adult (4-month-old) p62^P394L+/+^ male mice and WT littermates (Daroszewska et al., 2011). In view of the p62 P394L mutation-induced potentiation of age-related increase in osteoclastogenesis *in vitro*, we asked whether the p62 P394L mutation had an *in vivo* effect on age-related bone loss. We examined the distal femoral metaphyses of 12-month-old p62^P394L+/+^ mice and WT littermates using micro computed tomography (μCT). There was a significant decrease in bone volume to total volume (BV/TV) of 33% (*P*<0.01), a significant decrease in trabecular number (Tb.N) and a significant increase in trabecular separation (Tb.Sp) in aged p62^P394L+/+^ mice compared with WT littermates (Fig. 2), in keeping with accelerated age-related bone loss. There were no significant differences in vertebral (L5) morphometry between p62^P394L+/+^ mice and WT littermates (data not shown).

### Evolution of pagetic-like lesions with ageing in p62^P394L^ mutant mice

**Fig. 3.**
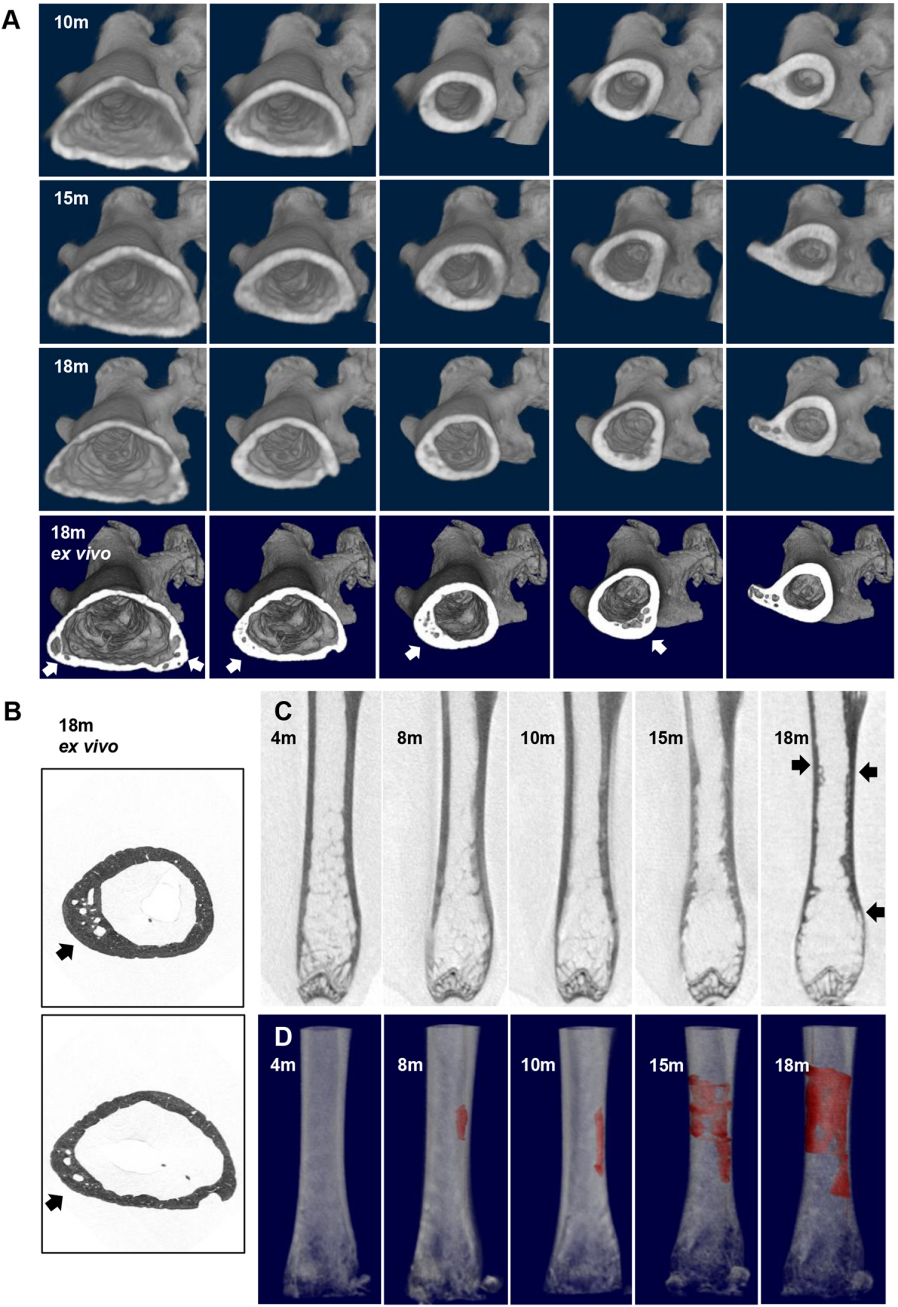
**Pagetic-like lesion evolution in the p62^P394L+/+^ mouse.** (A) A female p62^P394L+/+^, PBS-treated mouse was scanned *in vivo* with μCT at 18 μm resolution, as shown, until 18 months of age (top three rows) and an *ex vivo* scan was then performed at 9 μm resolution (bottom row). Cross-sections of 3D reconstruction starting just above the femoral condyles and ending at the trochanter are shown to visualise the asymmetrical involvement of the femoral shaft and progression over time. The lesion details are better seen on the *ex vivo* scan (bottom row, arrows). (B) To assess the lesion in greater detail, an *ex vivo* 1.5 μm scan was performed (arrows point to the lesion, which is also seen in the bottom row of A in the second and third panel from the left). (C) Longitudinal views of the femoral shaft and the lesion progression and extent (arrows). (D) The lesion was first observed at 8 months and was coloured in red for better identification and calculation of the rate of progression. Note the difference in orientation: in panels A and B versus C and D the hip is on the right- versus left-hand side, respectively. Owing to movement artefacts, the 12 month scan is not shown. m, age in months.

In the PBS-treated group, 8/10 (80%) p62^P394L+/+^ mice developed pagetic-like lesions in the femur and/or tibia with the morphology described before (Daroszewska et al., 2011) by 12 months, as compared with 0/10 (0%) in the ZA-treated p62^P394L+/+^ mice (*P*<0.001, Fisher's exact test; see also next section). As femoral pagetic-lesions in patients progress linearly at an estimated rate of 9.4 mm per annum (Renier and Audran, 1997), we monitored PBS-treated p62^P394L+/+^ mice *in vivo* with µCT to capture and follow up lesion progression. An example of the most severe lesion observed in this cohort and its evolution until the age of 18 months is shown in Fig. 3. The linear progression (Fig. 3D) between the age of 8 and 10 months was from 1.173 to 2.304 mm (change of 1.131 mm); between 10 and 15 months from 2.304 to 4.146 mm (change of 1.842 mm); and between 15 and 18 months from 4.146 to 4.696 mm (change of 0.55 mm). Thus, the average linear progression rate was 0.37 mm per month (4.47 mm per year) to involve ∼28.5% of the femur, given the femoral length of 16.5 mm, and the lesion gradually expanded in 3D as well (Fig. 3). Given that mice over 6 months old age 25× faster than humans (www.jax.org), and that a female human femur is, on average, 445 mm long (human femur length to mouse femur length, 445 mm/16.5 mm=26.97), the 1.131 mm change over 2 months in mouse is estimated to correspond to a 7.42 mm change per annum in a human. Likewise, the 1.842 mm (over 5 months) and 0.55 mm (over 3 months) changes in mice correspond to 4.84 mm and 2.41 mm growth per annum, respectively, in a human. Accordingly, the average mouse lesion progression rate of 4.47 mm per year corresponds to a 4.89 mm annual progression in human.

### Effect of ZA on the development of pagetic-like lesions and bone morphology

**Fig. 4.**
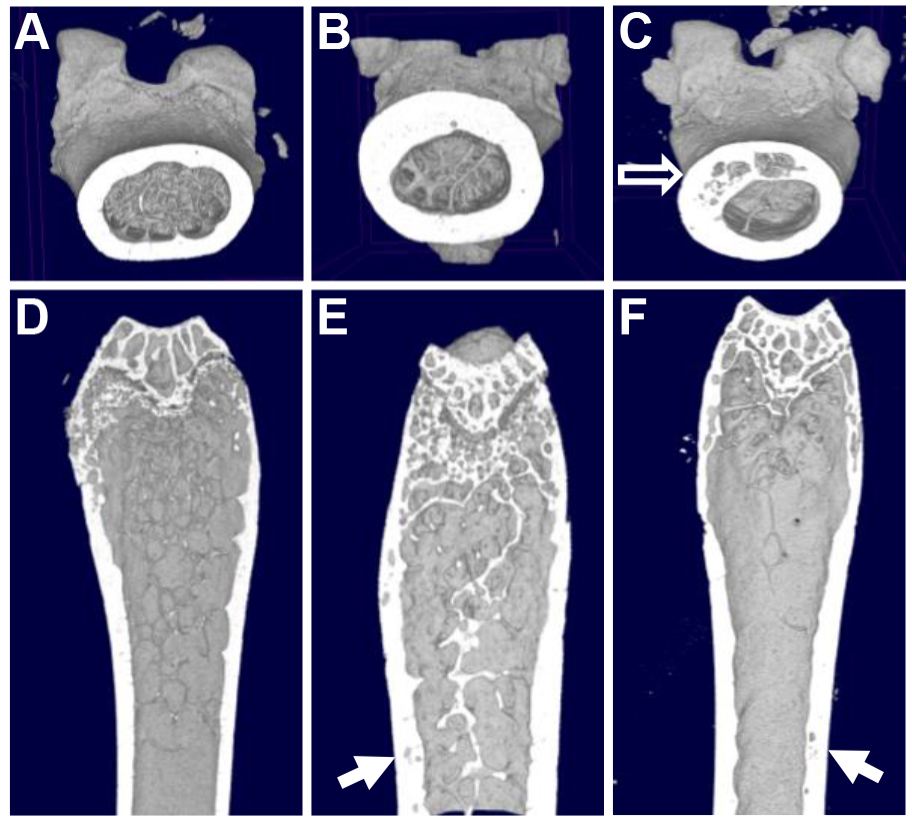
**Effect of ZA on the p62^P394L+/+^ mice bone phenotype analysed by μCT.** 12-month-old female p62^P394L+/+^ mice were treated with ZA or PBS from the age of 4 months. (A) μCT 3D reconstruction in transverse view of an untreated WT mouse. (B) μCT 3D reconstruction in transverse view of the p62^P394L+/+^ mouse treated with ZA; significant cortical thickening is evident. (C) μCT 3D reconstruction in transverse view of the p62^P394L+/+^ mouse treated with PBS; a pagetic-like lesion is indicated (empty arrow). (D) μCT 3D reconstruction in longitudinal view of an untreated WT mouse. (E) μCT 3D reconstruction in longitudinal view of the p62^P394L+/+^ mouse treated with ZA; cylindrical shape, preservation of trabeculae and cortical thickening are evident, and intracortical lucencies are indicated (arrow). (F) μCT 3D reconstruction in longitudinal view of the p62^P394L+/+^ mouse treated with PBS; the section includes the edge of the pagetic-like lesion indicated by the arrow. Representative 3D reconstructions of femurs are shown. PBS-treated p62^P394L+/+^ mice, *n*=7; ZA-treated p62^P394L+/+^ mice, *n*=9; WT untreated mice, *n*=6.

To clarify whether BPs could prevent the development of pagetic-like lesions in the p62^P394L+/+^ mice, we chose ZA, which is a widely used treatment for PDB and osteoporosis. We monitored for lesion development using *in vivo* μCT at 1-2 month intervals, but did not detect any pagetic-like lesions in either of the p62^P394L+/+^ groups treated with ZA (hence the data were pooled). However, a significant change in bone morphology was observed in all ZA-treated p62^P394L+/+^ compared with vehicle-treated p62^P394L+/+^ and untreated WT mice (Fig. 4). The cortex was significantly thickened in the ZA-treated p62^P394L+/+^ mice (Fig. 4B) compared with untreated WT mice, and more so when compared with the vehicle-treated p62^P394L+/+^ mice affected by pagetic-like lesions (where cortical thickening is part of the picture; Fig. 4C). The metaphyses shape in the ZA-treated p62^P394L+/+^ mice was cylindrical as opposed to flute-like in the vehicle-treated p62^P394L+/+^ and untreated WT mice (Fig. 4D-F). The growth plate looked highly mineralised. Trabeculae were very well preserved and plate-like as opposed to rod-like structures seen with ageing (Fig. 4E). Although no obvious pagetic-like lesions in the ZA-treated p62^P394L+/+^ mice occurred, we observed lucencies in the significantly thickened cortex (Fig. 4E, compare with F).

**Fig. 5.**
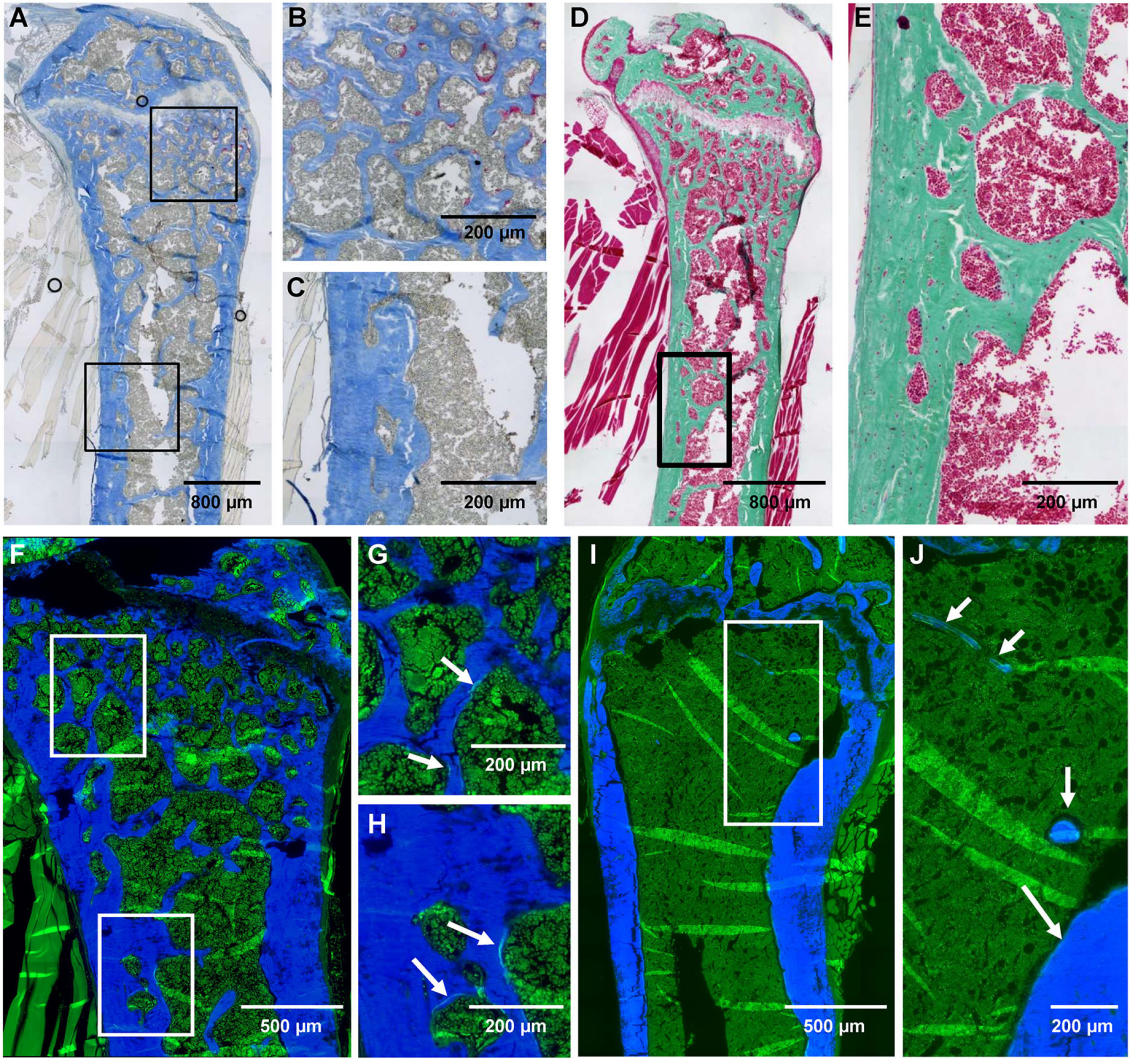
**Effect of ZA on the** **p62^P394L+/+^ mice bone phenotype analysed by histology.** (A-C) Aniline Blue and TRAcP stain of a femur from a ZA-treated p62^P394L+/+^ mouse. The top box in A delineates the part near the growth plate, where osteoclasts are clearly seen in red (B). The bottom box in A delineates part of the cortex, where lucencies filled with bone marrow are present, but no osteoclasts are seen (C). (D) Goldner's trichrome stain of a femur from a ZA-treated p62^P394L+/+^ mouse with no visible osteoid seams. (E) Higher magnification view of the cortex delineated by the box in D. (F-H) Fluorescent microscopy of a femur from a ZA-treated and calcein double-labelled p62^P394L+/+^ mouse, with Calcein Blue counterstain; very little, mostly single, calcein labelling is visible. The top box in F delineates the part near the growth plate shown at higher magnification in G. The bottom box in F delineates part of the cortex, shown at higher magnification in H. Arrows in G and H point to single calcein label. (I,J) Fluorescent microscopy of a femur from a PBS-treated and calcein double-labelled p62^P394L+/+^ mouse, with Calcein Blue counterstain; double labelling is seen (arrows). Note that the double labelling is seen on the few trabeculae present in the PBS-treated p62^P394L+/+^ mouse compared with significantly less and mostly single labelling seen in the ZA-treated p62^P394L+/+^ mouse, despite significantly higher trabecular bone volume in the latter.

On histology examination, tartrate resistant acid phosphatase (TRAcP)-stained osteoclasts were easily seen near the growth plate of ZA-treated p62^P394L+/+^ mice (Fig. 5A,B); however, no osteoclasts were identified within the lucencies of the thickened cortex (Fig. 5A,C). Thus, treatment with ZA did not inhibit all osteoclastogenesis, which was ongoing near the growth plate. The cortical lucencies are unlikely to represent treated pagetic-like lesions, as no disorganised bone or osteoclasts were evident (Fig. 5A,C). Goldner's trichrome stain showed no osteoid seams (Fig. 5D,E), and very little calcein double label was present (Fig. 5F,H) by 12 months in ZA-treated p62^P394L+/+^ mice, suggesting suppressed new bone formation. There was very little label in the cortical lucencies (Fig. 5F,H) [increased labelling would be expected in active pagetic-like lesions (Daroszewska et al., 2011)]. Bone histomorphometry analysis revealed significantly increased bone volume per tissue volume (BV/TV) in ZA-treated versus PBS-treated p62^P394L+/+^ mice (Table S1). However, bone formation parameters – mineral apposition rate (MAR), mineralising surface per bone surface (MS/BS) and bone formation rate per bone surface (BFR/BS) – were significantly reduced in the ZA- compared with PBS-treated p62^P394L+/+^ mice (Table S1).

**Fig. 6.**
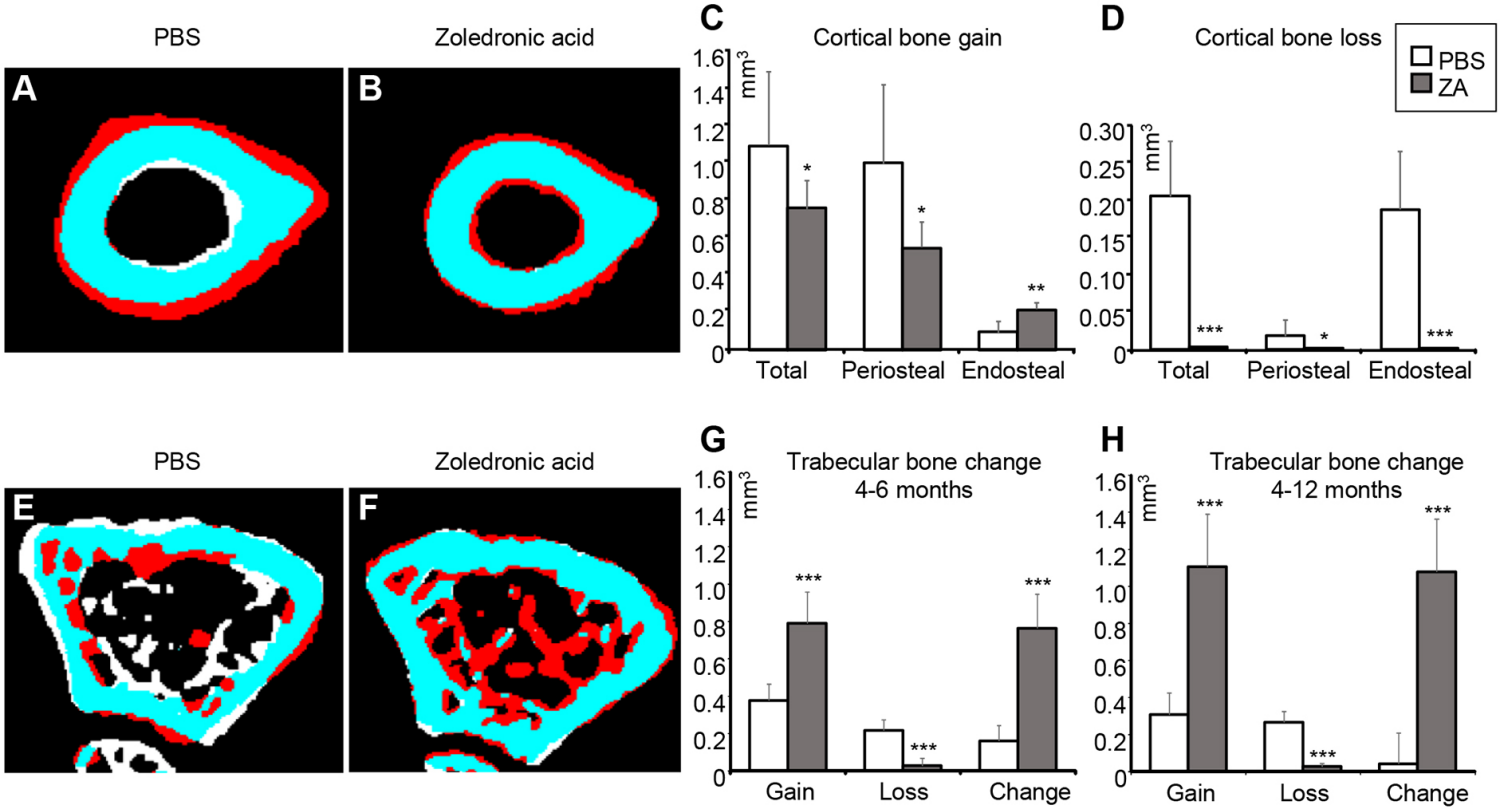
**Effect of ZA on the bone shape of the p62^P394L+/+^ mice analysed by μCT.** ZA- or PBS-treated p62^P394L+/+^ mice were scanned using a Skyscan 1076 *in vivo* µCT scanner (resolution 18 µm) at 4, 6 and 12 months of age. The scans were registered and femoral bone shape changes over time were analysed. (A,B,E,F) White indicates bone lost, red indicates bone gained and blue indicates no change between the time points. A and B show changes at the mid diaphysis between 4 and 12 months of age. PBS-treated p62^P394L+/+^ mice show endosteal bone loss and periosteal bone gain (A), whereas ZA-treated mice show both endosteal and periosteal bone gain and very little bone loss (B). E and F show changes in the distal femoral metaphysis between 4 and 6 months of age. Note the substantial increase in bone volume in the ZA-treated animal (F). (C,D) Quantification of changes in A and B. (G) Quantification of the trabecular bone volume changes between 4 and 6 months of age. (H) Quantification of the trabecular bone volume changes between 4 and 12 months of age. Data are mean±s.d. *P<0.05, **P<0.01, ***P<0.001 (Student's *t*-test). PBS, *n*=6; ZA, *n*=8. Anchor points added by the CTAn software have been removed from panels A, B, E and F.

We then analysed the dynamic bone morphometry of the ZA- and PBS-treated p62^P394L+/+^ mice using the obtained *in vivo* µCT scans. Between 4 and 12 months of age, the PBS-treated p62^P394L+/+^ mice lost endosteal, while gaining periosteal, bone at the femoral mid-shaft, which led to an increase in bone diameter and marrow space (Fig. 6A,C,D). In contrast, the ZA-treated mice gained both endosteal and periosteal bone, although the periosteal bone gain was reduced compared with that of PBS-treated control animals (Fig. 6B-D). Treatment with ZA led to a rapid increase in trabecular bone volume during the first 2 months (0.38±0.09 mm^3^/month; Fig. 6F,G), owing to almost complete suppression of bone resorption and a substantial increase in bone formation. Over the next 6 months, the bone volume further increased, but at a much slower rate (0.05±0.04 mm^3^/month, *P*<0.001; Fig. 6E-H). The PBS-treated control p62^P394L+/+^ animals showed a small increase in trabecular bone volume; however, this was partially offset by trabecular bone loss at 6 months (net bone gain between 4 and 6 months, 0.08±0.04 mm^3^/month; Fig. 6E,G), and completely offset by 12 months of age (net bone gain between 6 and 12 months, −0.02±0.03 mm^3^/month; Fig. 6H).

### ZA increases bone mass in p62^P394L+/+^ mice

**Fig. 7.**
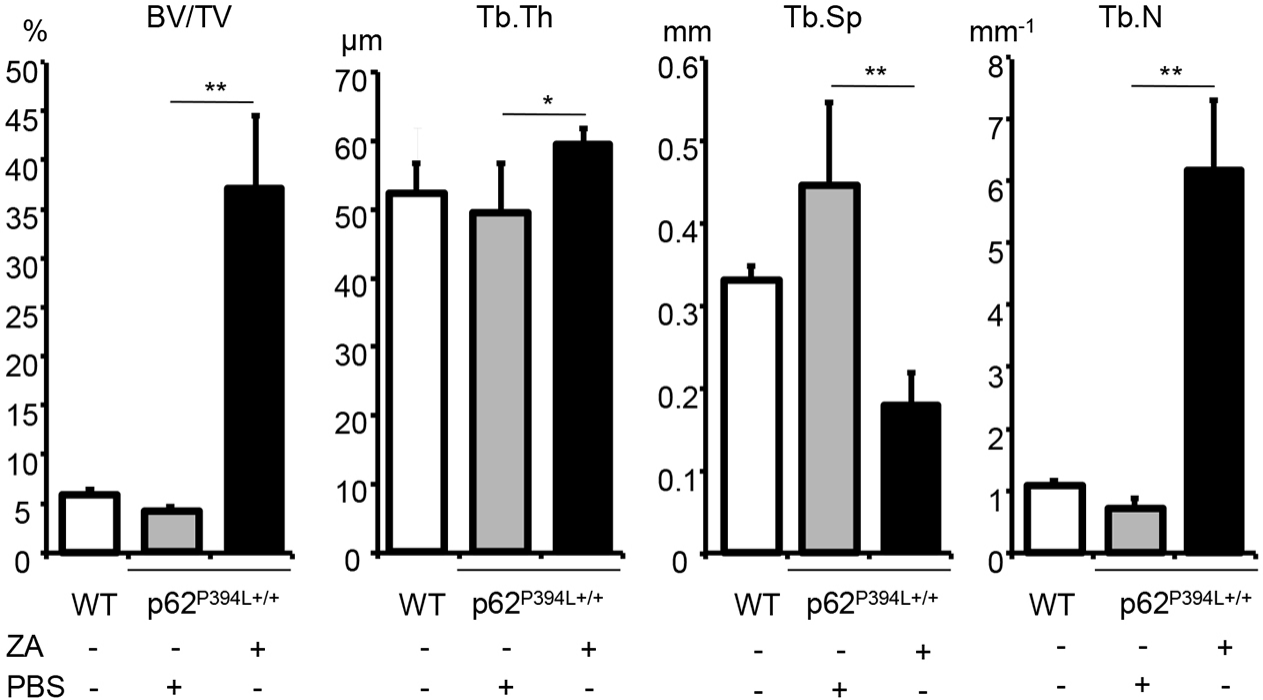
**ZA increases bone volume in p62^P394L+/+^ mice.** 4-month-old female p62^P394L+/+^ mice were treated with ZA (*n*=9) or PBS (*n*=7) as indicated until the age of 12 months. WT mice (*n*=6) were untreated. Bone morphometry of femoral metaphyses was carried out by μCT at 4.5 μm resolution. BV/TV, bone volume per tissue volume; Tb.Th, trabecular thickness; Tb.Sp, trabecular separation; Tb.N, trabecular number. Data are mean±s.d. *P<0.05, **P<0.01 (one-way ANOVA).

In view of the striking changes in the ZA-treated p62^P394L+/+^ mice bone morphology, we carried out a μCT morphometry analysis. At 12 months, BV/TV was approximately tenfold higher in the ZA- compared with PBS-treated p62^P394L+/+^ mice (*P*<0.001; Fig. 7), in keeping with histomorphometry findings (Table S1). Tb.Th increased by 20% (*P*<0.01), Tb.Sp decreased by 60% (*P*<0.001) and Tb.N increased by over sixfold (*P*<0.01) in the ZA-treated group (Fig. 7). There was no significant difference between the four mice that received six doses, and the six mice that received five doses, of ZA; therefore, the data were pooled. Thus, ZA not only prevented the development of pagetic-like lesions and protected against the accelerated bone loss in the p62^P394L+/+^ mice, but substantially increased bone volume and enhanced bone structure.

### Long-term treatment with ZA leads to highly mineralised bone in the p62^P394L+/+^ mice

**Fig. 8.**
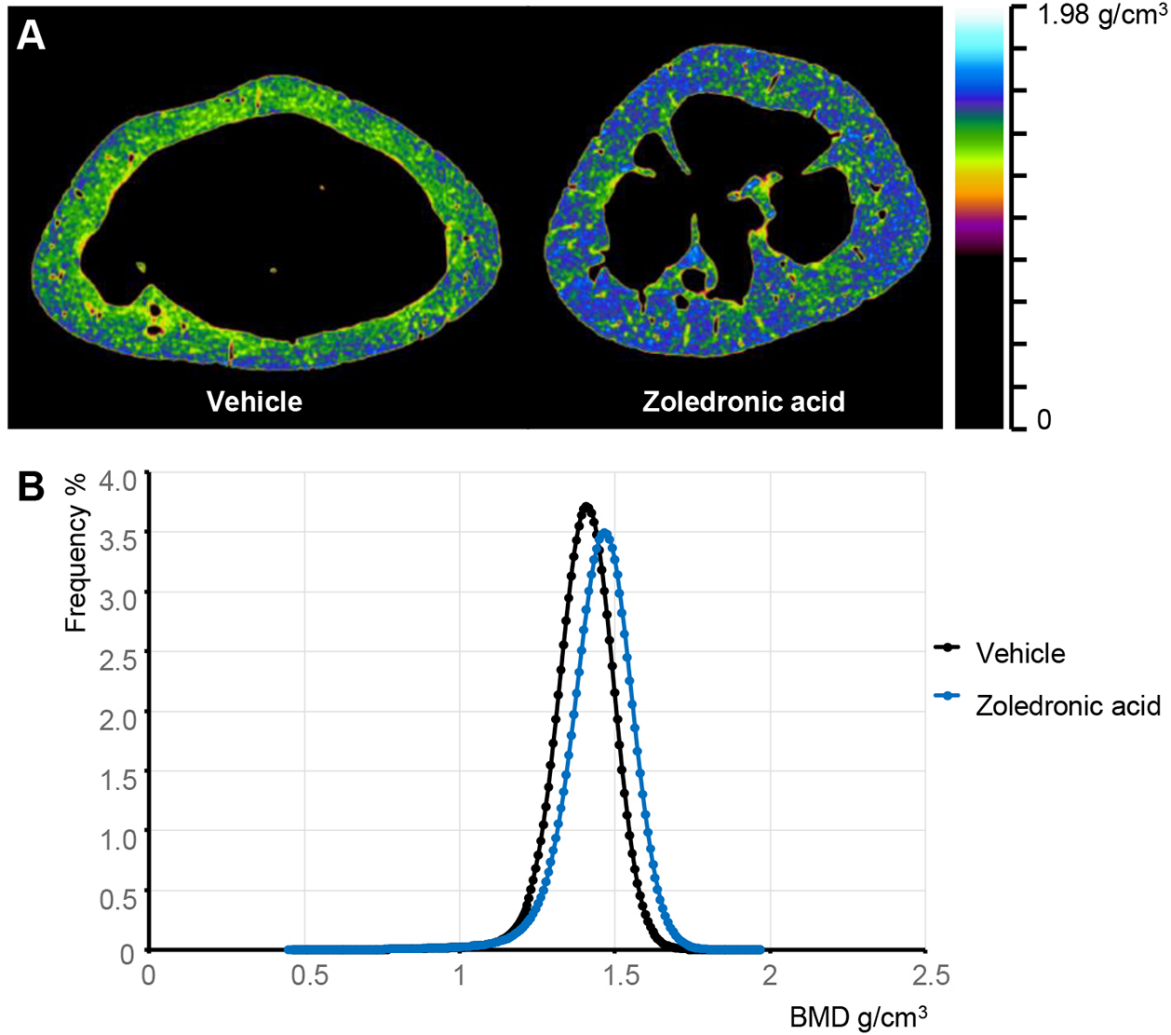
**ZA increases bone mineralisation in p62^P394L+/+^ mice.** 4-month-old female p62^P394L+/+^ mice were treated with ZA (*n*=8) or vehicle (PBS, *n*=7) at 2 month intervals until the age of 12 months. Bone mineralisation was assessed at the same level in the diaphysis of the femoral bones. (A) The ZA-treated mice have highly mineralised bones compared with the vehicle-treated controls. (B) The bone tissue mineral density (TMD) is significantly higher in the ZA-treated mice compared with controls. The graph shows the density distribution within the bone tissue. The samples from ZA-treated mice show significantly higher mean density, and a widening of the density distribution.

Long-term administration of ZA to young adult p62^P394L+/+^ mice led to suppression of bone turnover, which can result in hypermineralisation of bone matrix (Allen and Burr, 2011). Furthermore, during sectioning for histology, we observed that the bone samples were brittle and caused damage to the microtome knives, suggesting high mineralisation. To investigate whether the bones of ZA-treated mice were indeed highly mineralised, we performed additional scans of the distal femurs, with increased averaging and camera binning to reduce image noise, and analysed tissue mineralisation. The ZA-treated p62^P394L+/+^ mice had significantly higher bone tissue mineralisation compared with the PBS-treated p62^P394L+/+^ mice (Fig. 8A). The mean bone mineralisation density in the ZA-treated cohort was 1.47±0.031 g/cm^3^, compared with 1.416±0.032 g/cm^3^ in the PBS-treated cohort (*P*<0.01). There was also an increased width of the density distribution in the treated group (standard deviation of the distribution 0.105±0.004 g/cm^3^) compared with that in the control group (standard deviation of the distribution 0.093±0.004 g/cm^3^; *P*<0.001) (Fig. 8B).

## DISCUSSION

We have previously shown that the p62 P394L mutation is sufficient to induce a pagetic-like phenotype in mice, characterised by focal, asymmetric, mixed osteolytic/osteosclerotic lesions predominantly affecting the long bones, femur and tibia, with increased penetrance with ageing (Daroszewska et al., 2011). We also demonstrated the presence of microtubular structures in osteoclasts within PDB-like lesions similar to those previously reported in human PDB (Daroszewska et al., 2011). In the present study, we examined the effect of the p62 P394L mutation on age-related osteoclastogenesis and bone loss, the development and progression of pagetic-like lesions, and the prophylactic use of ZA.

It is well established that osteoclasts and osteoclast precursors from patients with PDB, with or without the p62 P392L mutation, are hypersensitive to RANKL (Chamoux et al., 2009; Menaa et al., 2000; Neale et al., 2000). We have previously shown that osteoclast precursors generated from the bone marrow of 3-month-old p62^P394L+/−^ and p62^P394L+/+^ mice were hypersensitive to RANKL, and that both heterozygous and homozygous osteoclasts showed higher bone resorption than the WT (Daroszewska et al., 2011). Here, we have extended our analysis to assess the effect of ageing on p62^P394L^ osteoclast RANKL hypersensitivity, and found that it increased incrementally in all groups, i.e. WT, heterozygotes and homozygotes, with a p62 P394L allele dose effect. The *in vitro* findings of an age-related increase in RANKL-induced osteoclast formation in WT concur with previous findings in mice (Cao et al., 2005; Perkins et al., 1994) and humans (Chung et al., 2014; Koshihara et al., 2002). The role of p62 in osteoclastogenesis has been established by Duran and colleagues, who reported that targeted disruption of p62 in mice impaired osteoclastogenesis mediated by PTHrP (also known as PTHLH) (Duran et al., 2004), whereas overexpression of the human p62 P392L mutation in murine osteoclasts has been shown to increase osteoclastogenesis and induce a low bone mass phenotype in young adult mice, which progressed with ageing (Kurihara et al., 2007). Human studies have shown that the p62 P392L variant increased osteoclast differentiation, nuclearity and longevity (Chamoux et al., 2009). However, our finding of age-related enhancement of already high RANKL-induced osteoclast formation owing to the p62 P394L mutation (with an allele dose effect) is novel, and suggests that the p62 P394L mutation significantly potentiates osteoclastogenesis in an otherwise already pre-osteoclast-rich ageing skeleton (Farr et al., 2017), which intuitively might be permissive for relatively minor stimuli to induce further ‘uncontrolled’ osteoclastogenesis and pagetic lesions. In line with these observations, the known age-related increase of constitutive RANKL expression in stromal cells, osteoblasts and osteocytes (Cao et al., 2005; Chung et al., 2014; Piemontese et al., 2017) could be a contributory factor to the development of an osteoclast formation-permissive environment (Hiruma et al., 2008).

As age-related bone loss coincides with increased osteoclast activity, which is potentiated by the p62 P394L mutation, we hypothesised that the p62 P394L mutation would cause accelerated bone loss with ageing. We previously showed that long bone morphometry of young adult (4-month-old) p62^P394L+/+^ mice was no different to WT (Daroszewska et al., 2011). In a previous study of the equivalent to our p62^P394L+/+^ transgenic mouse, Hiruma and colleagues did not see any morphometric differences in the spine for up to 12 months of age (Hiruma et al., 2008), and we similarly did not find evidence of increased bone loss at the spine in the present study (data not shown). However, we found a significant increase in bone loss of the hind limb bones of p62^P394L+/+^ mice by 12 months of age, which suggests that the p62 P394L mutation enhances age-related bone loss, which likely becomes first apparent in the long bones, as age-related bone loss takes place in the long bones ahead of the vertebrae in mice (Glatt et al., 2007). Interestingly, pagetic-like lesions also develop preferentially in the long bones (Daroszewska et al., 2011) rather than vertebrae (Daroszewska et al., 2011; Hiruma et al., 2008), which raises the possibility that biomechanical factors might interact with the p62 P394L mutation to influence where and when bone lesions develop. Locomotion differences between human (bipedal) and mouse (quadripedal) carry different mechanical loading, which coincides with differences in pagetic lesion distribution: axial skeleton and the long bones are preferentially affected in human, whereas long bones (but not the axial skeleton) are preferentially affected in mice. Interestingly, development of pagetic lesions in humans has been described in bones subject to decades of supraphysiological repetitive mechanical loading (Gasper, 1979; Solomon, 1979). Animal work has shown that mechanical loading-induced bone fatigue or microfractures (Cardoso et al., 2009; Noble et al., 2003; Verborgt et al., 2000, 2002) led to apoptosis of osteocytes (key mechanosensing cells in bone), which occurs focally. As osteocyte apoptosis promotes focal osteoclast activation (Kogianni et al., 2008), it is possible that in the ageing skeleton affected by the p62 P392/4L mutation, highly primed for RANKL-mediated osteoclastogenesis, a trigger for focal pagetic lesion development could be localised osteocyte apoptosis; for example, due to microcracks developing with ageing, or repetitive mechanical loading-induced bone fatigue. This hypothesis warrants further investigation because, if confirmed, it could have significant translational implications for carriers of the p62 P392L mutation and affected individuals.

In terms of mechanisms underlying the increased age-related RANKL-induced osteoclast formation potentiated by the p62 P392/4L mutation, it is possible that alterations in the autophagy pathway play a role. Whilst DeSelm and colleagues provided evidence for the noncanonical role of autophagy in the resorptive function of osteoclasts (DeSelm et al., 2011), we have previously shown increased expression of key regulatory autophagy genes – *SQSTM1*, autophagy-related gene-5 (*ATG5*) and microtubule-associated light chain 3 (*LC3*; also known as *MAP1LC3A*) – as well as increased accumulation of LC3-II after treatment with bafilomycin in pre-osteoclasts and osteoclasts, respectively, generated from young adult p62^P394L+/+^ mice compared with WT mice (Daroszewska et al., 2011), in keeping with induction of the autophagy pathway. However, the effect of ageing on canonical and noncanonical autophagy pathways in osteoclasts is currently unknown.

The finding of the p62 P394 mutation's ability to accelerate age-related RANKL hypersensitivity of osteoclasts, paralleled by increased bone loss in p62^P394L+/+^ mice, is also interesting from the translational perspective. Although *in vitro*, the p62^P394L+/+^ osteoclasts showed the highest RANKL hypersensitivity, heterozygous osteoclast formation was also significantly increased compared with WT, implying that p62^P394L+/−^ mice could also show accelerated bone loss with ageing. Indeed, we have previously shown an allele dose effect of the p62 P394L mutation on the PDB-like phenotype severity (Daroszewska et al., 2011) and, as such, focused our investigations on homozygotes. Thus, we have not aged heterozygotes in the current study in an effort to use the minimum number of mice necessary in keeping with the principles of the 3Rs (Replacement, Reduction and Refinement of Animals in Research; https://nc3rs.org.uk). As the vast majority of patients with the p62 P392L-associated PDB are heterozygous, although rare homozygous or compound heterozygous cases with severe disease have been reported (Collet et al., 2007; Eekhoff et al., 2004; Laurin et al., 2001, 2002; Morissette et al., 2006), it is unclear whether our findings could be translated to a potentially increased risk of osteoporosis in the carriers of the p62 P392L mutation. Intriguingly, whilst PDB is classically considered a focal disease, there is evidence of increased bone remodelling in sites unaffected (Meunier et al., 1980), and possibly an increased risk of vertebral and rib fractures, again at unaffected sites (Melton et al., 2000). However, whether patients with PDB, p62 P392L mutation-linked PDB or unaffected mutation carriers are at an increased risk of osteoporosis is currently unknown.

We have previously shown that the PDB-like phenotype penetrance in the p62^P394L+/+^ mice increased with ageing and reached 70% and 95% by 8 and 12 months, respectively (Daroszewska et al., 2011). Here, we were interested to capture the moment of the PDB-like lesion occurrence and progression over time. Using an *in vivo* μCT approach, we confirmed that the lesions had mixed osteolytic/osteosclerotic morphology and enlarged over time, but generally did not occur before the age of 6 months. We did not observe purely sclerotic lesions (to indicate ‘burned out’ PDB), which can occur in long-standing PDB in some patients (Siris et al., 1980); however, this is not unexpected given the higher rate of bone turnover in mice, compared with humans and a relatively short observation period, as our mice were culled at the age of 12-18 months. We have previously shown that the lesions most commonly developed at the distal femur and proximal tibia and less commonly in the shaft of the long bones (Daroszewska et al., 2011), which is comparable with a similar distribution in humans in the long bones, albeit with more predilection for the proximal ends of the long bones in the latter (Renier et al., 1996). Here, we presented a lesion in the diaphysis of the femur, which progressed over time. The estimated rate of its linear progression was equivalent to 7.42 mm per annum growth in human at early stages and later to between 4.84 mm and 2.41 mm per annum, which compares to an ∼9.4 mm per annum linear progression of a femoral pagetic lesion in human, according to estimations made two decades ago (Renier and Audran, 1997), when PDB was more severe than currently. Thus, although the murine pagetic-like lesions appear to progress at a slower rate than the human ones, we argue that their progression is comparable. Indeed, the slowing down of progression with ageing, arguably could suggest eventual ‘burning out’ of the phenotype.

There is evidence that treatment with BPs inhibits PDB lesion progression and promotes formation of histologically normal bone (Reid et al., 1996). BPs also suppress the raised bone turnover that is characteristic of active PDB, and ZA is the most potent BP (Corral-Gudino et al., 2017). Thus, we were interested to assess whether ZA could prevent the development of pagetic-like lesions in our mouse model. No lesions were detected in the ZA-treated p62^P394L+/+^ mice, providing evidence of the efficacy of this agent in suppressing the raised bone turnover that occurs in this mouse model of PDB. Furthermore, the effectiveness of ZA given early, prior to lesion development, provides pre-clinical evidence, to support the approach used in the ZiPP study (ISRCTN11616770; https://doi.org/10.1186/ISRCTN11616770), in which ZA is being investigated as a means of preventing development of PDB lesions in *SQSTM1* mutation carriers.

Although studying the general skeletal effect of ZA was not the primary objective of our experiments, our study led to a number of important observations with potential translational implications. The positive effect of ZA on bone volume was striking, and ZA had a profound inhibitory effect on bone resorption. Although the brittle nature of the sections from ZA-treated mice precluded formal measurement of bone resorption parameters by histomorphometry, observation of the (mostly badly damaged) sections indicated a significant reduction in osteoclast numbers in 12-month-old ZA-treated p62^P394L+/+^ mice. Moreover, ZA attenuated new bone formation, but with a net effect of increased bone volume. Furthermore, bones of the ZA-treated p62^P394L+/+^ mice showed significantly higher mineralisation compared with controls, which suggests that ZA significantly slowed down the bone remodelling process, which allowed for increased mineralisation over time. On the other hand, the change of shape at the mid-femoral shaft consequent to treatment with ZA suggests that modelling was still taking place, although it was impaired. The dose-dependent protective effect of ZA on bone has been described previously in animal models of ovariectomy-induced osteoporosis (Gasser et al., 2008). ZA is commonly used for treatment of both PDB and osteoporosis, and long-term use of BPs has been associated with an increased risk of atypical fractures (Schilcher et al., 2011), believed to be facilitated by the oversuppression of bone turnover (Compston, 2011); however, we did not observe any fractures in any of the mice by 12 months of age. We also did not see any evidence of microfractures on high resolution 2.5 μm scans (*n*=9, data not shown). Nevertheless, we were unable to formally assess bone strength, as the above observations were made on already fixed bone samples analysed by µCT and histology. Regardless, the ability of ZA to increase bone mass and promote mineralisation (with potential consequences of accumulation of microdamage) with impairment of remodelling and, to a degree, modelling might have translational implications when choosing this type of treatment for young adult individuals, especially for prolonged periods of time, as already implied by this known effect of BPs (Allen and Burr, 2011). Thus, given incomplete penetrance and declining prevalence and severity of PDB, careful consideration would have to be given when contemplating prophylactic use of ZA in susceptible individuals, in the light of potential negative ramifications of prolonged suppression of bone remodelling. It is expected that the outcome of the ZiPP trial will be instrumental in future clinical decision making.

The current study has confirmed our previous findings that the p62 P394L mutation is sufficient to cause a PDB-like disorder in mice and has provided new insight into the pathophysiology of the increased age-related penetrance of this disorder. The increase in osteoclastogenic potential of bone marrow cells from p62^P394L^ mice with age provides an explanation for the increased penetrance with age that we previously observed in this model (Daroszewska et al., 2011), and for the increased penetrance of PDB with age in humans (Morissette et al., 2006). Furthermore, the preferential targeting of the lower limb bones with sparing of the vertebrae suggests that biomechanical factors could play a key role in determining where and when lesions develop (Daroszewska et al., 2011).

In summary, we have shown that osteoclastogenesis is enhanced with ageing and the p62 P394L mutation further increases age-related osteoclastogenesis and age-related bone loss. In the ageing skeleton, the p62 P394L mutation causes PDB-like lesions, which progress over time. It is unclear what triggers these lesions; however, the increased age-related osteoclastogenesis potentiated by the p62 P394L mutation seems to allow for lesion development, thus creating a permissive environment. ZA prevents the development of the PDB-like lesions, significantly increases bone volume and bone mineralisation, and interferes with age-related long bone shape changes.

## MATERIALS AND METHODS

### p62^P394L+/+^ mice

The p62^P394L+/+^ mice were generated by gene targeting as previously described (Daroszewska et al., 2011) and housed in a standard animal facility with free access to water and food. The study was conducted in accordance with institutional, national and European regulations of laboratory animal care and use, and approved by the Home Office (UK). The mice were on a mixed 129/sv and C57/BL6 background, and the colonies were maintained by breeding heterozygotes; WT, heterozygous and homozygous animals used in this study were littermates. As in this mouse model of PDB, there is no phenotypic difference between males and females (Daroszewska et al., 2011), for the assessment of ZA (Novartis) in prevention of pagetic-like lesion development, ten female p62^P394L+/+^ mice were randomly allocated to receive treatment with ZA in phosphate-buffered saline (PBS) at a dose of 85 µg/kg subcutaneously (equivalent to a human 5 mg/60 kg dose) as of 4 months of age, at which age mice are fully mature, but have no detectable pagetic-like lesions (Daroszewska et al., 2011). Of the ZA-treated p62^P394L+/+^ mice, four mice received three doses of ZA at monthly intervals and three subsequent doses at 2 month intervals (six doses in total). The remaining six mice received a total of five doses at 2 month intervals. The initial frequency of ZA administration at monthly intervals is approximately equivalent to 5 mg given at 2 year intervals to a human three times [given that a mouse ages 25× faster than a human (www.jax.org)]. Likewise, the frequency of administration of ZA 2 months apart to mice corresponds to 5 mg given 4.2 years apart to a human. This frequency corresponds broadly to the approach used in clinical practice for treatment of osteoporosis (three to six times annually) and PDB (repeat treatment depending on response). Ten control female p62^P394L+/+^ mice were randomly allocated to receive PBS alone at the same time points. These numbers were arrived at assuming that ZA would reduce the proportion of mice with lesions from 95% [as previously reported (Daroszewska et al., 2011)] to 19% by 12 months with a power of 80% and an α<0.05. ZA-treated mice were culled at 12 months. Most of the control PBS-treated p62^P394L+/+^ mice were culled at 12 months using cervical dislocation, but a small cohort was maintained until a maximum of 18 months. One of the control mice died after anaesthesia at 8 months and was replaced by an additional mouse.

### Cell culture

Bone marrow was obtained from 3- and 12-month-old mice. Osteoclasts were generated by treatment of macrophages with M-CSF (Prospec Bio) and RANKL (kindly donated by Dr Jim Dunford, University of Oxford, Oxford, UK), according to standard methods as previously described (Idris et al., 2008). RANKL dose response was performed in 96-well plates. Numbers of osteoclasts were counted after TRAcP staining by an observer blinded to genotype and mouse age.

### μCT analysis

The skin was removed and hind limbs were fixed in 4% formalin-buffered saline and stored in 70% ethanol. μCT analysis was performed using a Skyscan 1272 or the *in vivo* Skyscan 1076 system. For assessment of bone morphometry, femurs and the lumbar spine were dissected free of most soft tissue, and scanned at a resolution of 4.5 µm (60 kV, 150 µA, using a 0.5 mm aluminium filter). Samples with pagetic-like lesions in the distal femur were excluded from the morphometry analysis. The reconstruction was performed using the Skyscan NRecon package. Trabecular bone parameters were measured using Skyscan CTAn software in a stack of 200 slices immediately proximal to the growth plate as described (van ‘t Hof, 2012). To screen for the presence of lesions and follow up lesion progression, mice were scanned *in vivo* at 18 µm resolution at 1-2 month intervals between the age of 4 and 12 months (and at age 15 and 18 months where indicated) under general anaesthesia using halothane. Assessment of lesions was performed by an observer blinded to genotype and treatment allocation after reconstruction as described (Daroszewska et al., 2011). Skyscan dataviewer software was used to register the series of *in vivo* scans for assessment of lesion evolution and bone changes over time. Bone changes over time were analysed in 100 slices at the mid shaft of the femur for cortical bone analysis, and in 100 slices at the distal femoral metaphysis, for trabecular bone changes using macros in CTAn. In the cortex, any bone changes in contact with the marrow space in the 4 month scans were designated as endosteal, whereas changes in contact with the periosteal outline of the 4 month scans were designated as periosteal. For analysing trabecular changes, the macro used first separated the trabecular from the cortical compartment in 4 month scans, and subsequently measured the bone gain and loss in the trabecular compartment by subtracting the registered scans for the 6 and 12 month time points.

Finally, to analyse tissue mineralisation, samples were equilibrated overnight in water, and the distal femur scanned inside drinking straws using a Skyscan1272 scanner (resolution 4.5 µm, X-ray source at 50 kV and 200 µA, 0.5 mm Al filter, camera binning 2×2, rotation step size 0.3°, averaging at 3). Hydroxy-apatite standards (Skyscan), were scanned using identical settings to calibrate mineral density. Next, datasets consisting of 300 slices at the mid shaft of the femur were thresholded to identify bone, and the binary was eroded (3D space) with a sphere with a radius of 2 to remove voxels affected by partial voxel effects. This binary was then used as a mask to measure the mineral density in mineralised tissue only [the tissue mineral density (TMD)].

### Bone histology

Bone samples were processed and stained for histology as described by van ‘t Hof et al. (2017). Briefly, animals received calcein intraperitoneal injections 4 days and 1 day before culling. The skin was removed, hind limbs were fixed for 24 h in 4% formalin and stored in 70% ethanol. The samples were embedded in methyl methacrylate and 5 µm sections were cut using a tungsten steel knife. Sections were stained for TRAcP to visualise osteoclasts and counterstained with Aniline Blue. For analysis of calcein double labelling, sections were counterstained with Calcein Blue and histomorphometry performed as described by van ‘t Hof et al. (2017). All sections were visualised on a Zeiss Axioimager fluorescence microscope fitted with a QImaging Retiga 4000R digital camera. The histology assessment was performed by an observer blinded to genotype and treatment allocation.

### Statistical analysis

Statistical analyses were performed using SPSS version 21. Differences between genotype or treatment groups were determined by ANOVA, Student's *t*-test or Fisher's exact test. All data are presented as means±s.d. unless stated otherwise.

## Supplementary Material

Supplementary information
