# Supplementary material for: Zoledronic acid prevents pagetic-like lesions and accelerated bone loss in the p62P394L mouse model of Paget's disease
Source: Dis Model Mech. 2018 Aug 23;11(9):dmm035576. doi: 10.1242/dmm.035576 (PMC6177010; doi:10.1242/dmm.035576)
Supplement: Supplementary information [file dmm-11-035576-s1.pdf]

Table S1

Bone histomorphometry of zoledronic acid-treated versus vehicle-treated p62$^{P394L+/+}$ mice.

	BV/TV (%)	MAR (µm/day)	MS/BS (%)	BFR/BS (µm^3/µm^2/day)
PBS	4.53±3.06	1.13±0.16	28.36±20.69	0.35±0.27
ZA	39.38±16.91	0.83±0.10	6.13±3.84	0.05±0.03
p	<0.001	<0.01	<0.05	<0.05

Histomorphometry was performed on distal femurs of p62$^{P394L+/+}$ mice treated with zoledronic acid (ZA, N=5) or vehicle (PBS, N=9). Values are means and standard deviations. BV/TV: bone volume per tissue volume; MAR: mineral apposition rate; MS/BS: mineralising surface per bone surface; BFR/BS: bone formation rate per bone surface.
